# Utilisation of the Hip Disability and Knee Injury Osteoarthritis Outcome Score in physiotherapy following total hip and knee arthroplasty: a cross-sectional survey

**DOI:** 10.1080/21679169.2024.2421821

**Published:** 2024-11-03

**Authors:** Dennis J. van den Berg, Henri Kiers, Esther T. Maas, Thea P. M. Vliet Vlieland, Raymond W. J. G. Ostelo

**Affiliations:** a Department of Health Sciences, Faculty of Science, Vrije Universiteit Amsterdam, Amsterdam Movement Sciences, Amsterdam, The Netherlands; b Institute for Movement Studies, University of Applied Sciences Utrecht, Utrecht, The Netherlands; c Dutch Association for Quality in Physiotherapy, Zwolle, The Netherlands; d Department of Orthopaedics, Rehabilitation and Physiotherapy, Leiden University Medical Centre, Leiden, The Netherlands; e Basalt Rehabilitation, Leiden, The Netherlands; f Department of Health, University of Applied Sciences Leiden, Leiden, The Netherlands; g Department of Epidemiology and Data Science, Amsterdam UMC, Location Vrije Universiteit, Amsterdam Movement Sciences Research Institute, Amsterdam, The Netherlands

**Keywords:** Hip arthroplasty, knee arthroplasty, patient outcome assessment, patient reported outcome measures, physiotherapy

## Abstract

**Objective:**

To explore the frequency of administration and the usage of the Hip Disability and Knee injury Osteoarthritis Outcome Scores (HOOS/KOOS) and their Physical function Short forms (HOOS-PS/KOOS-PS) by physiotherapists after total hip and knee arthroplasties (THA/TKA).

**Design:**

A cross-sectional study using an open online survey.

**Setting:**

Primary care physiotherapy practices affiliated with the Dutch Association for Quality in Physiotherapy.

**Participants:**

Physiotherapists with experience treating over five patients with a THA or TKA within the past 5 years.

**Results:**

One hundred and sixty-six physiotherapists completed the survey (median age: 40.0 years, female: 34%, median experience: 15.0 years). Of those, 32 did not administer the HOOS(-PS) or KOOS(-PS) (‘non-users’), 41 administered only due to organisational requirements or guideline recommendations (‘passive users’) and 93 actively used them for individual patient treatment purposes (‘active users’). ‘Treatment evaluation’, ‘diagnosis’, and ‘prognosis’ were most often reported as potential reasons to actively use the HOOS(-PS) or KOOS(-PS) for individual treatment purposes. Determinants associated with active use of the HOOS(-PS) or KOOS(-PS) appeared to be fewer years of experience as a physiotherapist, a larger treatment volume of THA/TKA, a younger age, and higher attitude scores regarding PROM use.

**Conclusions:**

Most responding physiotherapists administer the HOOS(-PS) or KOOS(-PS), but their use for individual treatment is limited. Active users appear to be less experienced, younger, treat larger volumes of THA/TKA, and possess a more positive attitude towards using patient-reported outcome measures.

## Abbreviations

HOOS: Hip Disability and Osteoarthritis Outcome Score
KOOS: Knee injury and Osteoarthritis Outcome Score
HOOS-PS: Hip Disability and Osteoarthritis Outcome Score-Physical function Short form
KOOS-PS: Knee injury and Osteoarthritis Outcome Scores-Physical function Short form
TKA: Total knee arthroplasty
THA: Total hip arthroplasty
PROMs: Patient-reported outcome measures
LEFS: Lower Extremity Functional Scale
OHS: Oxford Hip Score
OKS: Oxford Knee Score
WOMAC: Western Ontario and McMaster University Osteoarthritis Index
SKF: Dutch Association for Quality in Physical Therapy

## Introduction

Value-based healthcare aims to create value for patients by improving health outcomes from the patient’s perspective [1]. These health outcomes can be measured with patient-reported outcome measures (PROMs), which capture the experience of a patient’s health status [2]. The routine use of PROMs is considered an important characteristic of patient-centeredness and can potentially improve individual patient care [3–5]. Consequently, PROMs are internationally recommended for physiotherapy care following total hip and knee arthroplasty (THA/TKA) [6–11], of which more than 2 million are performed annually worldwide [12,13].

While several studies explored the use of PROMs by physiotherapists [14–18], we identified only two studies that specifically reported on the use of PROMs by physiotherapists following THA and TKA [19,20]. In a survey involving 178 physiotherapists in Canada, findings revealed that the Lower Extremity Functional Scale (LEFS) was used by 59% of respondents for clinical decision-making and by 45% of respondents for program evaluation. Around 30% of the respondents incorporated the Oxford hip and knee score (OHS/OKS) into their clinical decisions, and approximately 25% used them for program evaluation. In contrast, less than 5% of the respondents utilised the Hip and Knee Osteoarthritis Outcome Score (HOOS/KOOS) for clinical decisions and program evaluation [19]. Another survey among physiotherapists in the United States showed that 76% of respondents reported having considerable experience with the LEFS for clinical decision-making. By contrast, this proportion was approximately 20% for the OHS/OKS, 25% for the HOOS and 15% for the KOOS. However, it is unlikely that the results of these two studies fully reflect the use of PROMs for individual patient care following total hip and knee replacement. Additionally, contextual factors such as national and international PROM recommendations, changes in physiotherapy education programs regarding PROM use, and policymakers’ interests differ between countries and change over time.

More recently, the use of recommended PROMs in the care of patients undergoing THA and TKA has been studied through a survey among 142 orthopaedic surgeons in the Netherlands [21]. While these PROMs are administered in 60% of patients [22], only 13% of the orthopaedic surgeons used PROMs during consultations for THA and TKA [21]. The main reasons for the low use of PROMs during consultations were poor accessibility to individual PROMs data, no added value of PROMs during consultations from the orthopaedist perspective, issues in the interpretation of PROM results, and a lack of evidence on the benefit for individual care [21]. The authors concluded that ‘despite policymakers’ intentions to incorporate the routine use of PROMs to assist individual care, it continues to present challenges in orthopaedic care’ [21]. Whether these challenges also extend to the routine use of PROMs for individual patient treatment by physiotherapists, who play an important role in the care following THA and TKA, remains unknown.

Physiotherapy guidelines for the management of THA and TKA vary in their recommendations on which specific PROMs to use following THA and TKA [6–11,23]. While the Western Ontario and McMaster University Osteoarthritis Index (WOMAC) [6,7,9,23] and the OHS/OKS [6,9,23] are commonly recommended, both PROMs require a licence for use. The HOOS/KOOS were developed as an extension of the WOMAC and are non-proprietary. Additionally, various national [9,11,23] and international [6,10] guidelines recommend the HOOS and KOOS or their Physical function Short form versions (HOOS-PS and KOOS-PS), for physiotherapy care following THA and TKA. But how, and how frequently, these PROMs are used is unknown. Therefore, this study aimed to explore (1) the frequency of administration of the HOOS, HOOS-PS, KOOS and KOOS-PS, which will be collaboratively referred to as HOOS(-PS) and KOOS(-PS); (2) the purpose of their use, and their application during treatment; and (3) determinants associated with their usage during individual patient treatment following post-hip and knee replacement physiotherapy in Dutch primary care.

## Methods

### Design

We performed a cross-sectional online survey study. The CHERRIES checklist was followed as a reporting guideline [24]. The study protocol received ethical approval from the Research Ethics Review Committee (BETHCIE) of the Faculty of Sciences, Vrije Universiteit Amsterdam (registration number: 2022.043).

### Setting and recruitment

We recruited primary care physiotherapists affiliated with the Dutch Association for Quality in Physical Therapy (SKF) experienced in post-THA or TKA care. Recruitment occurred between March 2023 and October 2023 via convenience sampling, which despite its limitations in generalisability, is considered an effective and pragmatic solution for online survey research [25]. Study invitations, with a link to the study survey, were emailed to approximately 700 SKF-registered primary care practices, representing over 50% of Dutch primary care physiotherapists. Additionally, a study invitation was sent via the staff online newsletter of the Institute for Movement Studies at the University of Applied Sciences Utrecht and via email to several regional collaboration networks focusing on post-THA and TKA physiotherapy care. Also, peer-to-peer recruitment was carried out by the research team using information letters with scannable barcodes (linked to the survey). Eligibility criteria were communicated in the information letter and potential participants assessed their own eligibility. Informed consent was obtained through the survey.

Eligibility criteria for study participation were:

- Member of the SKF;
- Experience ranging from a minimum of five THA and/or TKA cases in the last five years to an unlimited number of cases, to capture a broad range of experience. This approach allowed us to explore different patterns of usage among physiotherapists with varying levels of experience in treating patients with THA and TKA, including perspectives of relatively infrequent users.

### Survey design

The survey comprised four main sections: (1) we developed seven multiple-choice questions based on pilot testing and previous research regarding the usage of PROMs in primary care physiotherapy practices [15,16]. These questions addressed the frequency of administration, underlying reasons for usage, and applications of the HOOS(-PS) and KOOS(-PS). Three questions had an open-ended ‘other, please explain:’ category. (2) Twenty-seven statements regarding attitude (nine statements), knowledge (seven statements) and organisational context (eight statements) related to the use of PROMs in general, developed by Meerhoff et al. [16]. Agreement with these statements was scored on a five-point Likert scale ranging from ‘I completely disagree’ to ‘I completely agree’. The final scores for attitude, knowledge and context were computed by aggregating their respective questions resulting in ranges from 9 to 45, 7 to 35 and 8 to 40, respectively. Higher scores on these variables indicate a more favourable attitude towards PROM use, greater knowledge about PROMs, and a more supportive organisational environment for PROM use. (3) Six questions regarding participant characteristics (age, sex, work experience, region of employment, member of a collaboration network focusing on rheumatic diseases, additional education with regards to rheumatic diseases, and treatment volume of THA and TKA). (4) Two questions regarding primary care practice characteristics (full-time equivalents within the organisation and extra quality certification).

Pilot testing of the survey was done in three phases. First, the research team thoroughly reviewed the order, flow, skip logic and time to complete the survey. Second, two eligible participants were asked to think aloud while completing the survey. Finally, the survey was distributed among a small subset of potential participants. After each phase, changes to the total number of questions, skip logic, question formulation and answer categories were made. A full overview of the survey is shown in Appendix 1.

Surveys were completed online using the Qualtrics software package (Qualtrics, Provo, UT). Participants were informed about the study aims, duration, data security and anonymity via the survey’s introductory page. Adaptive questions were utilised to reduce the number and complexity of the questions. Forced response fields were used to improve questionnaire completeness, and Qualtrics settings prevented multiple survey completions from one device. Before submitting the survey, participants were able to review and change their answers through a back button. Surveys were anonymised without recording the IP address.

### Statistical analysis

Descriptive statistics were used to describe the frequency of administration of the HOOS(-PS) or KOOS(-PS), the reasons for use and their applications during treatment, physiotherapist characteristics and primary care practices characteristics for the overall sample, ‘passive-users’, ‘active users’ and ‘non-users’.

To explore factors associated with the usage of the HOOS(-PS) and KOOS(-PS) for individual treatment purposes, respondents were divided into ‘passive-users’, ‘active users’ and ‘non-users’ based on whether they administered the HOOS(-PS) or KOOS(-PS) and on the underlying reasons for administering these questionnaires. To distinguish users who merely administered the HOOS(-PS) or KOOS(-PS) from those who actively use these PROMs for individual treatment purposes, a question about the underlying reasons for administering the HOOS(-PS) or KOOS(-PS) was included. Those who exclusively administered the HOOS(-PS) or KOOS(-PS) due to organisational requirements or guideline recommendations were categorised as ‘passive-users’. Respondents who additionally indicated at least one of the following reasons: ‘diagnosis’, ‘treatment evaluation’, ‘prognosis’, ‘clinical decision-making’, ‘patient education’, or ‘shared decision-making’ were classified as ‘active users’. Respondents indicating to not administer the HOOS(-PS) or KOOS(-PS) at all were classified as ‘non-users’.

Statistical analyses were conducted utilising custom scripts developed in Python (version 3.9.13). The Pandas, NumPy and SciPy libraries were used to facilitate data manipulation, numerical computations and statistical processing, respectively.

## Results

### Recruitment flow

A total of 234 physiotherapists responded to the survey. Out of these, 47 respondents did not agree to participate in the study. After excluding 21 surveys with no responses to any questions, we were able to include 166 surveys, yielding a completion rate of 89% (166/187).

### Physiotherapist and primary care practice characteristics

Participant demographics are summarised in Table 1. The median age of the participants was 40.0 (interquartile range (IQR): 30.5–54.0) years and 34% (56/166) of respondents were female. The median number of full-time equivalents for primary care practices was 8.0 (IQR: 4.5–16.0).

### Frequency of HOOS(-PS) and KOOS(-PS) administration

Table 2 provides a summary of how often HOOS(-PS) and KOOS(-PS) are administered, the underlying reasons for administering them, and their application during treatment. Out of the 166 respondents, 134 reported administering the HOOS(-PS) or KOOS(-PS), with the majority (104/134, 78%) administering the HOOS(-PS) and KOOS(-PS) in 50% or more of their patients. The HOOS(-PS) and KOOS(-PS) were most frequently administered at the beginning (117/134, 87%) and the end (108/134, 81%) of the treatment, while they were less frequently administered during the treatment period (67/134, 50%).

### Reasons for administering the HOOS(-PS) and KOOS(-PS) and their application

Among the 134 respondents who administered the HOOS(-PS) or KOOS(-PS), 41 (31%) indicated that they administered them solely because of organisational requirements or guideline recommendations, categorising them as ‘passive users’. The remaining 93 (69%) reported at least one of the following reasons: ‘diagnosis’, ‘treatment evaluation’, ‘prognosis’, ‘clinical decision-making’, ‘patient education’, or ‘shared decision-making’ or ‘other’ as their main purpose(s) for administering the HOOS(-PS) or KOOS(-PS) and were categorised as ‘active users’.

Among the 93 active users, 80 (86%) reported using the HOOS(-PS) or KOOS(-PS) in 50% or more of patients. Most active users (78/93, 84%) reported treatment evaluation as their main application of the HOOS(-PS) or KOOS(-PS). In contrast, 36 (39%) or fewer active users reported using them for diagnosis, prognosis, patient education, shared decision-making and clinical decision-making.

The impact of the HOOS(-PS) and KOOS(-PS) use on clinical decision-making was relatively limited, with most active users (79/93, 85%) reporting that these PROMs ‘only sometimes’, ‘seldom’ or ‘never’ influenced their clinical decisions. In contrast, 63 (68%) active users reported that they ‘often’ or ‘always’ engaged HOOS(-PS) or KOOS(-PS) outcomes in the discussion with their patients, while only 26 (28%) active users reported discussing these outcomes with their professional peers.

### Frequency of and reasons for not administering the HOOS(-PS) or KOOS(-PS)

Thirty-two respondents indicated not to administer the HOOS(-PS) or KOOS(-PS), categorising them as ‘non-users’. The most frequently mentioned reasons for not administering the HOOS(-PS) or KOOS(-PS), ‘No value for clinical decision-making’ (12/32, 38%) and ‘Other’ (10/32, 31%). Within the ‘Other’ category, the perceived irrelevance of the questions for the patient population was most frequently mentioned (6/32, 19%).

### Determinants associated with active or passive HOOS(-PS) or KOOS(-PS) use

Active users were, on average, 9 years younger than passive users, with a median age of 36.0 (IQR: 30.0–51.0) compared to 45.0 (IQR: 34.5–53.8), respectively. Additionally, active users had on average, 8.5 years less work experience compared to passive users, with a median work experience of 17.0 years (IQR: 6.0–23.0) compared to 21.5 years (IQR: 8.0–29.5), respectively. Notably, 60% of active users (56/93) treated 11 or more patients with THA and TKA each year, whereas only 32% of passive users (12/32) treated the same volume of patients. Additionally, active users scored higher in attitude (mean difference: 7.9, SD: 1.1), Knowledge (mean difference: 2.8, SD: 0.7) and Organisational context scores (median difference: 3.0) regarding PROMs. Primary care practices of active users were on average 3.0 full-time equivalents larger compared to passive users, with a median size of 9.0 (IQR: 4.5–20.0) compared to 6.0 (4.0–15.0), respectively.

## Discussion

This study showed that 81% of the responding physiotherapists administer the HOOS(-PS) or KOOS(-PS) following THA and TKA, while 56% actively utilise them for individual patient treatment purposes, primarily for treatment evaluation. Active users of the HOOS(-PS) or KOOS(-PS) appear to be younger, have fewer years of experience as physiotherapists, treat larger volumes of patients following THA and TKA, and have a more positive attitude towards the use of PROMs compared to passive users.

In our study, 56% of physiotherapists actively use the HOOS(-PS) or KOOS(-PS), rates notably higher compared to Canada (<5%) [19] and the US (<30%) [20]. This higher usage is likely due to familiarity with these PROMs, the inclusion of the shorter HOOS-PS and KOOS-PS versions and national guideline recommendations. Similar explanations were offered for higher LEFS use in Canada [19] and the US [20]. HOOS(-PS) and KOOS(-PS) use by our respondents mirrored LEFS use in Canada (59%) but were lower compared to LEFS use in the US (77%). However, there are uncertainties regarding the interpretation of the US and Canada survey results. Both surveys included limited applications of PROMs (clinical decision-making [19,20] and treatment evaluation [19]), which raises the question of whether physiotherapists using these PROMs for other purposes classified themselves as ‘experienced’ or not. Additionally, ‘experience’ may not necessarily reflect actual usage. Our study is distinctive in that it offers an overview of the frequency of administration of the HOOS(-PS) and KOOS(-PS); distinguishes between passive-users, active users and non-users; and more comprehensively explores HOOS(-PS) and KOOS(-PS) applications for individual treatment purposes.

We also explored determinants associated with HOOS(-PS) or KOOS(-PS) usage, as was done for other PROMs [15,16]. Experience, both as a physiotherapist as well as with the target population, was also linked to PROM usage in another study. The study showed that experience with the target population was positively associated with usage of the STarT Back Screening Tool, but negatively associated with usage of the Quebec Back Pain Disability Scale (QBPDS) [15]. Additionally, QBPDS usage was negatively associated with experience as a physiotherapist. These findings suggest that experienced physiotherapists may be more critical in selecting PROMs. Another explanation might be that more experienced physiotherapists integrate the content of PROMs into their patient history taking, thereby avoiding the need to administer PROMs. Unlike our findings, another study found no link between attitudes towards PROMs and their usage [16]. However, this study did not differentiate between administering PROMs and using them in treatment, suggesting that attitudes may affect PROM use in treatment rather than their administration.

Most active users indicated using the HOOS(-PS) or KOOS(-PS) for treatment evaluation. This may be due to the Dutch physiotherapy guideline for osteoarthritis of the hip and knee, which recommends these PROMs for treatment evaluation [11]. Additionally, the guideline provides minimal clinically important differences for the full HOOS and KOOS, which can be used for treatment evaluation [11]. However, they are not provided for the HOOS-PS and KOOS-PS, yet 73% of users reported using these shorter versions. The shorter length of the HOOS-PS and KOOS-PS likely contributes to their higher utilisation, as this has shown to be an important factor for PROM use by physiotherapists [5]. Moreover, most PROMs, including the HOOS(-PS) [26,27] and the KOOS(-PS) [28,29] have primarily been developed for group-level research [30] and may be less reliable for individual patients [31]. This may explain the limited impact of these PROMs use on clinical decision-making reported by our sample.

Despite recent attention within national [32,33] and international [34] literature towards the use of PROMs for shared decision-making, our results demonstrated limited use for this application. This may be due to the unavailability of normative population scores for the HOOS(-PS) and KOOS(-PS), which are considered ‘essential to the paradigm shift in utilising PROMs for clinical decision-making’ and are used to demonstrate patients’ expected progress and aid them in treatment selection [35]. However, the use of PROMs for shared decision-making, in the absence of normative population scores, has been demonstrated [36]. The use of a survey limited our exploration of how physiotherapists use the HOOS(-PS) or KOOS(-PS) for shared-decision making. Future qualitative research on physiotherapists’ experiences with the HOOS(-PS) or KOOS(-PS) for individual patient treatment, may provide valuable insight to increase their use in this context.

### Strengths and limitations

The strengths of this study include the comprehensive design of the survey, which incorporates insights from previous research and underwent multiple testing phases. Our exclusive focus on the HOOS(-PS) and KOOS(-PS) for physiotherapy following THA and TKA, could be seen as a limitation, however, it enabled us to thoroughly explore their usage. The restriction to SKF-registered physiotherapists might overestimate the actual use of HOOS(-PS) or KOOS(-PS), as these physiotherapists may be more engaged in quality improvement. While the regional distribution of our sample (North: 12%, East: 27%, South: 15% and West: 46%) resembled that of physiotherapists across the Netherlands (North: 9%, East: 19%, South: 22% and West: 49%) [37], the sample included a higher proportion of men (63%) compared to the national average (41%) [37]. Moreover, our results may not be generalisable to other countries, as healthcare systems differ between countries [38], which may influence PROM utilisation. Additionally, our survey was not formally validated. This lack of validation also pertained to the section of our survey based on Meerhoff et al.’s questionnaire [16]. While they employed a principal component analysis to determine attitude, knowledge and organisational context domains, the absence of formal validation is a limitation, as it is unknown whether these domains accurately reflect physiotherapists’ perspectives on PROM use.

### Implications for research and practice

Our results suggest that administering PROMs does not necessarily equate to using PROMs in daily clinical practice. Therefore, professional associations or other bodies requiring the administration of PROMs should not only focus on the administration of these measures but also on their usage during daily clinical practice. The underlying reasons behind the limited application of the HOOS(-PS) and KOOS(-PS) in daily clinical practice should be explored in future research. Moreover, our findings should be validated across countries with different healthcare systems.

In addition to the findings from our study, it is important to consider several general aspects when using PROMs as these are crucial in a broader context [39,40] even though they may not directly emerge from our research. More knowledge is needed on how to interpret absolute scores and changes in individual patients, as well as the potential implications for treatment. Similarly, understanding differences between practices at the group level is essential, as these may be influenced by various factors, including case mix variables.

This study has limited implications for clinical practice but identified potential determinants associated with PROM use in clinical practice, including age, experience as a physiotherapist, experience with THA and TKA, and attitude towards PROM use. These determinants may provide a basis for implementation initiatives.

## Conclusions

In conclusion, while the HOOS(-PS) and KOOS(-PS) appear to be frequently administered, their use for individual care appears to be limited. Physiotherapists using the HOOS(-PS) or KOOS(-PS) for individual treatment purposes appear to be younger, less experienced, treat larger volumes of patients following THA/TKA, and have a more positive attitude towards the use of PROMs compared to physiotherapists who administer the HOOS(-PS) or KOOS(-PS) solely due to organisational requirements or guideline recommendations.

## Supplementary Material

Appendix 1

## Figures and Tables

**Table 1:** Characteristics of 166 primary care physiotherapists responding to a survey on the use of the HOOS(-PS) or KOOS(-PS).

	Total sample^a^(*n* = 166)	Passive-usersb (*n* = 41)	Active usersc (*n* = 93)	Non-usersc (*n* = 32)
*Participant demographics*				
Age, median (IQR)	40.0 (30.5–54.0)	45.0 (34.5–53.8)	36.0 (30.0–51)	52.0 (34.5–53.8)
Sex, female, *n* (%)	56 (34)	13 (31)	34 (37)	9 (32)
Years of experience as physiotherapist, median (IQR)	15.0 (7.0–29.0)	21.5 (8.5–29.5)	13.0 (6.0–23.0)	21.5 (6.0–29.5)
Education level, *n* (%)				
Bachelor	83 (55)	17 (51)	52 (58)	14 (44)
Master	65 (43)	16 (46)	36 (41)	13 (41)
PhD	3 (2)	1 (3)	1 (1)	1 (3)
Region, *n* (%)				
North	18 (12)	5 (15)	8 (9)	5 (18)
East	41 (27)	10 (29)	25 (28)	6 (21)
South	23 (15)	5 (15)	14 (16)	4 (14)
West	69 (46)	14 (41)	42 (47)	13 (46)
Administration of HOOS/KOOS or HOOS-PS/KOOS-PS among active and passive users, *n* = 134 (81%)				
HOOS/KOOS	31 (23)	10 (24)	21 (23)	n/a
HOOS-PS/KOOS-PS	103 (77)	31 (76)	72 (77)	n/a
Member of regional or national collaboration network focusing on osteoarthritis, *n* (%)	24 (16)	3 (9)	14 (16)	7 (22)
Additional education with regards to hip/knee osteoarthritis, *n* (%)	28 (19)	7 (21)	16 (18)	5 (18)
Number of yearly treated patients with THA/TKA, *n* (%)				
1–5	32 (20)	11 (27)	9 (10)	12 (38)
6–10	52 (31)	17 (42)	28 (30)	7 (22)
11–20	41 (25)	7 (17)	28 (30)	6 (19)
>20	41 (25)	6 (15)	28 (30)	6 (19)
Attitude, knowledge and organisational context scores in relation to PROMs use				
Attitude score (range: 9–45), mean (SD)	28.9 (6.6)	23.6 (6.5)	31.5 (4.6)	27.8 (7.8)
Knowledge score (range: 7–35), mean (SD)	28.8 (3.6)	26.9 (3.7)	29.5 (3.1)	29.1 (4.0)
Organisational context score (range: 8–40), median (IQR)	35.0 (30.0–38.0)	33.0 (30.0–39.0)	36.0 (31–39.0)	34.0 (28.0–38.0)
*Primary care practice characteristics*				
Amount of FTE in physiotherapy practice, median (IQR)	8.0 (4.5–16.0)	6.0 (4.0–15.0)	9.0 (4.5–20.0)	7.5 (5.8–14.3)
Extra quality certification (*n*, %)	138 (91)	32 (94)	81 (91)	25 (78)

SD: standard deviation; IQR: interquartile range; THA: total hip arthroplasty; TKA: total knee arthroplasty; HOOS(-PS): Hip Disability and Osteoarthritis Outcome score (-Physical Functioning Shortform); KOOS(-PS): Knee Disability and Osteoarthritis Outcome score (-Physical Functioning Shortform); n/a: not available.

a Fifteen incomplete cases.

b Seven incomplete cases.

c Four incomplete cases.

**Table 2:** Frequency of administration of the HOOS(-PS) and KOOS(-PS), reasons for their use, and their application during treatment.

Survey item	Passive users^a^(*n =* 41)	Active user^a^(*n* = 93)
Estimated percentage of patients for whom the HOOS(-PS) or KOOS(-PS) are administered, *n* (%)		
1–25%	4 (10)	2 (2)
25–50%	11 (28)	10 (11)
50–75%	8 (21)	16 (17)
75–100%	16 (41)	64 (70)
Time point of questionnaire administration^b^, *n* (%)		
Pre-treatment	30 (73)	87 (94)
In-treatment	10 (24)	57 (61)
Post-treatment	23 (56)	85 (91)
Other	10 (24)	0 (0)
Reason for using the HOOS(-PS)/KOOS(-PS)^b^, *n* (%)		
For diagnosis	n/a	36 (39)
For treatment evaluation	n/a	78 (84)
For prognosis	n/a	33 (35)
For clinical decision-making	n/a	24 (26)
For patient education	n/a	21 (23)
For shared decision-making	n/a	28 (30)
Influence of HOOS(-PS)/KOOS(-PS) use on clinical decision-making, *n* (%)		
Never	21 (55)	4 (4)
Seldom	12 (31)	23 (25)
Sometimes	5 (13)	52 (56)
Often	0 (0)	13 (14)
Always	0 (0)	1 (1)
Frequency of discussing HOOS(-PS)/KOOS(-PS) results with patients, *n* (%)		
Never	12 (32)	1 (1)
Seldom	10 (26)	7 (8)
Sometimes	9 (24)	22 (24)
Often	5 (13)	35 (38)
Always	2 (5)	28 (30)
Use of HOOS(-PS)/KOOS(-PS) during peer consultation, *n* (%)	2 (5)	26 (28)

SD: standard deviation; *n*: number of observations; HOOS(-PS): Hip Disability and Osteoarthritis Outcome score (-Physical Functioning Shortform); KOOS(-PS): Knee Disability and Osteoarthritis Outcome score (-Physical Functioning.

a Three incomplete cases.

b Multiple answers possible.
