# Supplementary material for: Utilisation of the Hip Disability and Knee Injury Osteoarthritis Outcome Score in physiotherapy following total hip and knee arthroplasty: a cross-sectional survey
Source: Eur J Physiother. Author manuscript; Available in PMC 2024 Nov 15. (PMC7616814; doi:10.1080/21679169.2024.2421821)
Supplement: Appendix 1 [file EMS199843-supplement-_Appendix_1.pdf]

Survey HOOS(-PS)/KOOS(-PS) usage

Instructions: you may also use the HOOS/KOOS and HOOS-PS/KOOS-PS for other musculoskeletal conditions. For the purpose of this study, we would kindly ask you to relate the responses only to the use of the HOOS and KOOS during post-operative physiotherapy after **total hip or knee replacement.**

1 How many patients do you see per year for post-operative physiotherapy after a total hip or knee replacement?

- ○ 1-5
- ○ 6-10
- ○ 11-20
- ○ >20

2 Which questionnaire have you mainly used in the past 4 years during post-operative physiotherapy after a total hip or knee replacement?

- ○ HOOS/KOOS
- ○ HOOS-PS/KOOS-PS
- ○ Neither

'Skip To': 12 'If' Which questionnaire have you mainly used in the past 4 years during post-operative... = Neither

S.E.A. Beers
Sworn translator - Wbtv no. 29246

3 For what percentage of patients with a total hip and/or knee replacement do you typically administer the HOOS(-PS) or KOOS(-PS)?

○ 1 - 25%

○ 25 - 50%

○ 50 - 75%

○ 75 - 100%

---

4 When do you administer the HOOS(-PS) or KOOS(-PS)? (multiple answers possible)

☐ During the intake

☐ During interim evaluations

☐ At the end of the treatment

☐ Other, namely: ______________________________

S.E.A. Boers
Sworn translator - Wbtv no. 29246

5 What is the reason you have mainly used the HOOS(-PS) or KOOS(-PS)? (multiple answers possible)

- ☐ Obligation by health insurance company, Stichting Keurmerk Fysiotherapie, employer or other party
- ☐ Due to recommendation in KNGF guideline osteoarthritis hip and knee
- ☐ Diagnostics: assessing the severity and nature of symptoms
- ☐ Prognostic: estimating the course after total hip or knee replacement
- ☐ Evaluative: evaluation of treatment
- ☐ To support clinical decision-making
- ☐ To support the shared decision-making with the patient
- ☐ To support patient education
- ☐ Other, namely: ____________________

---

6 To what extent do outcomes of the HOOS(-PS) or KOOS(-PS) influence your clinical decision-making?

- ○ (Almost) always
- ○ Often
- ○ Sometimes
- ○ Rarely
- ○ Never

S.E.A. Boers
sworn translator - Wbtv no. 29246

7 To what extent do you discuss the outcomes of the HOOS(-PS) or KOOS(-PS) with your patients?

- ◯ Almost always
- ◯ Often
- ◯ Sometimes
- ◯ Rarely
- ◯ Never

---

8 Do you use the outcomes of the HOOS(-PS) or KOOS(-PS) during peer consultation (multiple answers possible)

- ☐ Yes, during consultation with fellow physiotherapists
- ☐ Yes, during consultation with orthopaedists.
- ☐ Yes, during consultation with general practitioners.
- ☐ Yes, during consultation with a colleague working in another discipline, namely:

____

- ☐ No, I do not use the outcomes of the HOOS(-PS) or KOOS(-PS) during peer consultations

---

S.E.A. Boers
Sworn translator - Wbtv no. 29246

9 To what extent do you use the outcomes of the HOOS(-PS) or KOOS(-PS) during peer consultations?

- ◯ Almost always
- ◯ Often
- ◯ Sometimes
- ◯ Rarely
- ◯ Never

'Display This Question':

'If' Which questionnaire have you mainly used in the past 4 years during post-operative... = Neither

10 What is the reason you did not use the HOOS/KOOS and HOOS-PS/KOOS-PS? (multiple answers possible)

- ◯ The questionnaires do not add value within my clinical decision-making process
- ◯ It takes too much time to administer these questionnaires
- ◯ I do not want to burden my patients with questionnaires
- ◯ I already have to administer too many other mandatory questionnaires
- ◯ Other, namely: ____

S.E.A. Boers
sworn translator - Wbtv no. 29246

The questions below relate not only to the HOOS/KOOS and HOOS-PS/KOOS-PS but to the use of Patient-Reported Outcome Measures (**PROMs**) in general

10-26 For each statement, indicate the extent to which you agree or disagree with the statement.

S.E.A. Boers sworn translator - Wbtv no. 29246

	Completely disagree	Disagree	Neutral	Agree	Completely agree
I know where to find PROMs	○	○	○	○	○
I am able to use PROMs with my patients	○	○	○	○	○
I am able to interpret PROMs correctly	○	○	○	○	○
When using the PROMs, there remains enough room for me to make my own assessments	○	○	○	○	○
I think that the use of PROMs contributes to physiotherapy diagnosis	○	○	○	○	○
I think that PROMs can contribute to treatment evaluation	○	○	○	○	○
I know how to apply PROMs within physiotherapeutic methodical practice	○	○	○	○	○
I think that the use of PROMs contributes to the quality of physiotherapy care provision	○	○	○	○	○
I think that it is important to record patient data objectively through PROMs	○	○	○	○	○
I think that working with PROMs does <u>not</u> take up too much of my time	○	○	○	○	○
My view is that most patients find that completing PROMs is <u>not</u> too time-consuming	○	○	○	○	○
The use of PROMs gives me plenty of room to include patient wishes	○	○	○	○	○
I would like to use PROMs (more) during physiotherapy after a total hip or knee replacement	○	○	○	○	○
I use the PROMs in daily practice	○	○	○	○	○

S.E.A. Boers
sworn translator - Wbtv no. 29246

I have experienced the added value of using PROMs	○	○	○	○	○
Patients cooperate in the use of PROMs	○	○	○	○	○
The use of PROMs is an established part of my physiotherapy methodical practice	○	○	○	○	○
I think that the use of PROMs can contribute to shared decision-making with patients	○	○	○	○	○
I think that the use of PROMs can contribute to better patient-therapist communication	○	○	○	○	○
I think that the use of PROMs can add value in interdisciplinary communication	○	○	○	○	○

S.E.A. Boers
Sworn translator - Wbtv no. 29246

27-38 For each statement, indicate the extent to which you agree or disagree with the statement.

	Completely disagree	Disagree	Neutral	Agree	Completely agree	N/A
In my practice, PROMs are available	○	○	○	○	○	○
PROMs are easy to find in the electronic patient record	○	○	○	○	○	○
Within our practice, there are agreements on the way of taking PROMs	○	○	○	○	○	○
The management supports staff in the use of PROMs. (In case you are a manager yourself, reflect on yourself)	○	○	○	○	○	○
The management itself uses PROMs. (In case you are a manager yourself, reflect on yourself)	○	○	○	○	○	○
The management requires staff to report digitally using PROMs. (In case you are a manager yourself, reflect on yourself)	○	○	○	○	○	○
Peer-physiotherapists in my practice also use PROMs. (In case there are no peer-physiotherapists, enter N/A)	○	○	○	○	○	○
In our practice, we have substantive consultations on the use of PROMs. (In case there are no peer-physiotherapists, enter N/A)	○	○	○	○	○	○
The use of PROMs is included in the policy of our practice	○	○	○	○	○	○
PROMs are sent to patients electronically where possible	○	○	○	○	○	○
PROMs are completed at the practice by the patient.	○	○	○	○	○	○
I always have PROMs completed by the patient themselves	○	○	○	○	○	○

S.E.A. Boers
Sworn translator - Wbtv no. 2924

39 Age

◯ Enter your age here, in years: ____

40 Sex

◯ Female

◯ Male

◯ Other

◯ I'd prefer not to say

41 Work experience as a physiotherapist

◯ Here, enter the number of years you have been working as a physiotherapist:
____

42 Level of education

◯ Bachelor

◯ Master

◯ PhD

S.E.A. Boers
Sworn translator - Wbtv no. 29246

43 Have you taken any additional in-service training or refresher courses in the field of rheumatic diseases?

- ○ No
- ○ Yes, namely: ____

44 Are you a member of a regional or national network in the field of rheumatic diseases?

- ○ No
- ○ Yes, namely: ____

45 What position(s) do you hold within the practice where you work? (multiple answers possible)

- ☐ Practice holder
- ☐ Partnership member
- ☐ Manager
- ☐ Employee - variable salary
- ☐ Employee - fixed salary

S.E.A. Boers
Sworn translator - Wbtv no. 29246

46 In which province is the practice where you work located?

- ◯ Groningen
- ◯ Friesland
- ◯ Drenthe
- ◯ Overijssel
- ◯ Flevoland
- ◯ Gelderland
- ◯ Utrecht
- ◯ North Holland
- ◯ South Holland
- ◯ Zeeland
- ◯ North Brabant
- ◯ Limburg

---

47 Total number of FTE employed in the practice (including administrative staff)

◯ Enter the number of FTE working in your practice:

____

S.E.A. Boers
Sworn translator - Wbtv no. 29245

48 Did the practice have additional quality certification between 2018 and 2021?

- ◯ Yes
- ◯ No

Translator's note:
The page numbers of this document are seemingly incorrect. This sworn translation reflects pages 1 through 13 of a survey on the use of the HOOS(-PS)/KOOS(-PS) questionnaire.

S.E.A. Boers
Sworn translator - Wbtv no. 29246

TRANSLATOR'S STATEMENT

I, Sebastiaan Eduard Antonius Boers, sworn as translator for the English language at the District Court of The Hague in the Netherlands, registered in the 'Register voor Beëdigde Tolken en Vertalers' (Register for Sworn Interpreters and Translators) under number 29246, hereby declare that the attached translation into English from Dutch as performed by me, to the best of my knowledge, accurately reflects the content and meaning of the Dutch original document, which original document is attached to this translation.

Signed in The Hague, 09 OKT. 2024

Sebastiaan Eduard Antonius Boers, Sworn (Dutch to English) Translator

Addr. Urgent Vertalen
Javastraat 72
2585 AS The Hague
The Netherlands

Applying changes or additions to the original document or the translation is not allowed and invalidates these documents. The falsification or forging of documents or the use of aforesaid falsified or forged documents is punishable by law.

Tel. +31 (0)70 778 58 30
E-mail info@urgentvertalen.nl
Web. https://urgentvertalen.nl/

Enquete HOOS(-PS)/KOOS(-PS) gebruik

Instructie: het kan zijn dat u de HOOS/KOOS en HOOS-PS/KOOS-PS ook gebruikt voor andere musculoskeletale aandoeningen. Voor het doel van deze studie willen we u vriendelijk verzoeken om de antwoorden enkel te relateren aan het gebruik van de HOOS en KOOS tijdens postoperatieve fysiotherapie na een **totale heup- of knieprothese.**

---

1 Hoeveel patiënten ziet u per jaar voor postoperatieve fysiotherapie na een totale heup- of knieprothese?

- ○ 1-5
- ○ 6-10
- ○ 11-20
- ○ >20

---

2 Welke vragenlijst heeft u voornamelijk in de afgelopen 4 jaar gebruikt tijdens de postoperatieve fysiotherapie na een totale heup- of knieprothese?

- ○ HOOS/KOOS
- ○ HOOS-PS/KOOS-PS
- ○ Geen van beide

Skip To: 12 If Welke vragenlijst heeft u voornamelijk in de afgelopen 4 jaar gebruikt tijdens de postoperatieve... = Geen van beide

---

3 Bij hoeveel procent van de patiënten met een totale heup- en/of knieprothese neemt u doorgaans de HOOS(-PS) of KOOS(-PS) af?

- ○ 1 - 25%
- ○ 25 - 50%
- ○ 50 - 75%
- ○ 75 - 100%

4 Wanneer neemt u de HOOS(-PS) of KOOS(-PS) af? (meerdere antwoorden mogelijk)

- ☐ Tijdens de intake
- ☐ Tijdens tussentijdse evaluaties
- ☐ Aan het einde van de behandeling
- ☐ Anders namelijk: ______________________________

5 Wat is de reden dat u de HOOS(-PS) of KOOS(-PS) voornamelijk heeft gebruikt? (meerdere antwoorden mogelijk)

- ☐ Verplichting door de zorgverzekeraar, Stichting Keurmerk Fysiotherapie, werkgever of andere partij
- ☐ Vanwege aanbeveling in KNGF-richlijn artrose heup en knie
- ☐ Diagnostiek: het beoordelen van de ernst en aard van symptomen
- ☐ Prognostisch: inschatten van het beloop na een totale heup of knie prothese
- ☐ Evaluatief: evaluatie van de behandeling
- ☐ Ter ondersteuning van de klinische besluitvorming
- ☐ Ter onderstuening van gezamenlijke besluitvorming met de patiënt
- ☐ Ter ondersteuning van patiënt educatie
- ☐ Anders namelijk: ______________________________

6 In welke mate beïnvloeden uitkomsten van de HOOS(-PS) of KOOS(-PS) uw klinische besluitvorming?

- ○ (Vrijwel) altijd
- ○ Vaak
- ○ Soms
- ○ Zelden
- ○ Nooit

7 In welke mate bespreekt u de uitkomsten van de HOOS(-PS) of KOOS(-PS) met uw patiënten?

○ Vrijwel altijd

○ Vaak

○ Soms

○ Zelden

○ Nooit

8 Gebruikt u de uitkomsten van de HOOS(-PS) of KOOS(-PS) tijdens intercollegiaal overleg (meerdere antwoorden mogelijk)

☐ Ja tijdens overleg met collega fysiotherapeuten

☐ Ja tijdens overleg met Orthopeden.

☐ Ja tijdens overleg met Huisartsen.

☐ Ja tijdens overleg met een collega's die werkzaam zijn in een andere discipline namelijk:

____

☐ Nee ik gebruik de uitkomsten van de HOOS(-PS) of KOOS(-PS) niet tijdens intercollegiaal overleg

9 In welke mate gebruikt u de uitkomsten van de HOOS(-PS) of KOOS(-PS) tijdens intercollegiaal overleg?

○ Vrijwel altijd

○ Vaak

○ Soms

○ Zelden

○ Nooit

Display This Question:

If Welke vragenlijst heeft u voornamelijk in de afgelopen 4 jaar gebruikt tijdens de postoperatieve... = Geen van beide

10 Wat is de reden dat u de HOOS/KOOS en HOOS-PS/KOOS-PS niet heeft gebruikt? (meerdere antwoorden mogelijk)

○ De vragenlijsten bieden geen meerwaarde binnen mijn klinische besluitvorming

○ Het kost te veel tijd om deze vragenlijsten af te nemen

○ Ik wil mijn patiënten niet belasten met vragenlijsten

○ Ik moet al te veel andere verplichte vragenlijsten afnemen

○ Anders namelijk:____

De onderstaande vragen hebben niet alleen betrekking op de HOOS/KOOS en HOOS-PS/KOOS-PS maar het gebruik van Patient gerapporteerde uitkomstmaten (**PROMs**) in het algemeen

10-26 Geef voor elke stelling aan in welke mate u het een of oneens bent met de stelling.

	Helemaal oneens	Oneens	Neutraal	Eens	Helemaal eens
Ik weet waar ik PROMs kan vinden	○	○	○	○	○
Ik ben in staat om PROMs te gebruiken bij mijn patiënten	○	○	○	○	○
Ik ben in staat om PROMs juist te interpreteren	○	○	○	○	○
Bij het gebruik van de PROMs blijft er voor mij voldoende ruimte om zelf afwegingen te maken	○	○	○	○	○
Ik vind dat het gebruik van PROMs een bijdrage levert aan het stellen van de fysiotherapeutische diagnose	○	○	○	○	○
Ik vind dat PROMs een bijdrage kunnen leveren aan de evaluatie van de behandeling	○	○	○	○	○
Ik weet hoe ik PROMs binnen het fysiotherapeutisch methodisch handelen kan toepassen	○	○	○	○	○
Ik vind dat het gebruik van PROMs een bijdrage levert aan de kwaliteit van de fysiotherapeutische zorgverlening	○	○	○	○	○
Ik vind het belangrijk om gegevens van patiënten objectief vast te leggen door middel van PROMs	○	○	○	○	○
Ik vind dat het werken met PROMs mij NIET te veel tijd kost	○	○	○	○	○
Mijn inschatting is dat de meeste patiënten vinden dat het invullen van PROMs NIET te veel tijd kost	○	○	○	○	○
Het gebruik van PROMs biedt mij voldoende ruimte om de wensen van de patiënt mee te laten wegen	○	○	○	○	○
Ik wil graag (meer) gebruik maken van PROMs tijdens fysiotherapie na een TKP of THP	○	○	○	○	○

Ik gebruik de PROMs in de dagelijkse praktijk	○	○	○	○	○
Ik heb ervaren dat het gebruik van PROMs een meerwaarde heeft	○	○	○	○	○
Patiënten werken mee aan het gebruik van PROMs	○	○	○	○	○
Het gebruik van PROMs is een vast onderdeel van mijn fysiotherapeutisch methodisch handelen	○	○	○	○	○
Ik vind dat het gebruik van PROMs kan bijdragen aan gezamenlijke besluitvorming met patiënten	○	○	○	○	○
Ik vind dat het gebruik van PROMs kan bijdragen aan betere patiënt-therapeut communicatie	○	○	○	○	○
Ik vind dat het gebruik van PROMs een meerwaarde kan hebben in interdisciplinaire communicatie	○	○	○	○	○

27-38 Geef voor elke stelling aan in welke mate u het eens of oneens bent met de stelling.

	Helemaal oneens	Oneens	Neutraal	Eens	Helemaal eens	N.V.T.
In mijn praktijk zijn PROMs beschikbaar	○	○	○	○	○	○
PROMs zijn eenvoudig te vinden in het elektronisch patiënten dossier	○	○	○	○	○	○
Binnen onze praktijk zijn er afspraken over de manier van afnamen van PROMs	○	○	○	○	○	○
De leiding ondersteunt de medewerkers in het gebruik van PROMs. (In dien je zelf leiding bent dan jezelf beoordelen)	○	○	○	○	○	○
De leiding maakt zelf gebruik van PROMs. (Indien je zelf leiding bent dan jezelf beoordelen)	○	○	○	○	○	○
De leiding vereist dat de medewerkers met PROMs digitaal verslag leggen. (Indien jezelf leiding bent dan jezelf beoordelen)	○	○	○	○	○	○
Ook de collega-fysiotherapeuten in mijn praktijk gebruiken PROMs.(Indien er geen sprake is van collega-fysiotherapeuten N.V.T. invullen)	○	○	○	○	○	○
In onze praktijk wordt inhoudelijk overlegd over het gebruik van PROMs. (Indien er geen sprake is van collega-fysiotherapeuten N.V.T. invullen)	○	○	○	○	○	○
Het gebruik van PROMs is opgenomen in het beleid van onze praktijk	○	○	○	○	○	○
PROMs worden waar mogelijk elektronisch naar patiënten verstuurd	○	○	○	○	○	○
PROMs worden op de praktijk door de patiënt ingevuld.	○	○	○	○	○	○
Ik laat PROMs altijd door de patiënt zelf invullen	○	○	○	○	○	○

39 Leeftijd

- ◯ Voer hier uw leeftijd in jaren in: ______________________________

40 Geslacht

- ◯ Vrouwelijk
- ◯ Mannelijk
- ◯ Anders
- ◯ Wil ik liever niet zeggen

41 Werkervaring als fysiotherapeut

- ◯ Voer hier het aantal jaar dat uw als fysiotherapeut werkzaam bent in:

____

42 Opleidingsniveau

- ◯ Bachelor
- ◯ Master
- ◯ PhD

43 Heeft u aanvullende bij- of nascholingen gevolgd op het gebied van reumatische aandoeningen?

○ Nee

○ Ja namelijk:____

44 Bent u lid van een regionaal of landelijk netwerk op gebied van reumatische aandoeningen?

○ Nee

○ Ja namelijk:____

45 Welke functie(s) vervult u binnen de praktijk waar u werkzaam bent? (meerdere antwoorden mogelijk)

☐ Praktijkhouder

☐ Maatschapslid

☐ Leidingevende

☐ Medewerker variabel salaris

☐ Medewerker vast salaris

46 In welke provincie bevindt de praktijk waar u werkzaam bent zich?

- ○ Groningen
- ○ Friesland
- ○ Drenthe
- ○ Overijssel
- ○ Flevoland
- ○ Gelderland
- ○ Utrecht
- ○ Noord-Holland
- ○ Zuid-Holland
- ○ Zeeland
- ○ Noord-Brabant
- ○ Limburg

47 Totaal aantal FTE werkzaam in de praktijk (inclusief administratief medewerkers)

○ Vul hier het aantal FTE in dat werkzaam is in uw praktijk:

____

48 Had de praktijk tussen 2018 en 2021 een aanvullende kwaliteitscertificering?

- ○ Ja
- ○ Nee
